# Access to early-phase clinical trials in older patients with cancer in France: the EGALICAN-2 study

**DOI:** 10.1016/j.esmoop.2022.100468

**Published:** 2022-05-06

**Authors:** C. Baldini, E. Charton, E. Schultz, L. Auroy, A. Italiano, M. Robert, E. Coquan, N. Isambert, P. Moreau, S. Le Gouill, C. Le Tourneau, Z. Ghrieb, J.J. Kiladjian, J.P. Delord, C. Gomez Roca, N. Vey, F. Barlesi, T. Lesimple, N. Penel, J.C. Soria, C. Massard, S. Besle

**Affiliations:** 1 Gustave Roussy, Université Paris-Saclay, Drug Development Department (DITEP), Villejuif; 2 Laboratory for Immunomonitoring in Oncology (LIO), University Paris-Saclay, Gustave Roussy Cancer Campus, Villejuif; 3 Human and Social Sciences Department, Centre Léon Bérard, Lyon; 4 CEPED (UMR 196), Université de Paris, IRD, Paris; 5 SESSTIM, Sciences Economiques & Sociales de la Santé & Traitement de l’Information Médicale, CANBIOS Team (Équipe Labellisée LIGUE 2019), Aix-Marseille University, INSERM, IRD, Marseille; 6 Université Grenoble Alpes, CNRS, Sciences Po Grenoble, Pacte, Grenoble; 7 Institut Bergonié, Early Phase Trials and Sarcoma Units, Bordeaux; 8 University of Bordeaux, Bordeaux; 9 Institut de Cancérologie de l’Ouest, Medical Oncology Department, Saint-Herblain; 10 François Baclesse Cancer Center, Department of Oncology, Caen; 11 Département d’Innovations Thérapeutiques/recherche Translationnelle en Oncologie et Hématologie (DITTOH), INSERM U1084, CHU de Poitiers, Poitiers; 12 Centre GF Leclerc, Medical Oncology Department, Dijon; 13 Service d’Hématologie Clinique, Unité d’Investigation Clinique, CHU, Nantes; 14 Department of Drug Development and Innovation, Institut Curie, Paris-Saclay University, Paris & Saint-Cloud; 15 APHP, Hopital Saint-Louis, Centre d’Investigations Cliniques, Paris; 16 Department of Medicine & Clinical Research Unit, Institut Claudius Regaud/Institut Universitaire du Cancer de Toulouse (IUCT-Oncopole), Toulouse; 17 Aix-Marseille University, CNRS, Inserm, Institut Paoli-Calmettes, Hematology Department, CRCM, Marseille; 18 Aix Marseille University, CNRS, INSERM, CRCM, APHM, Marseille Early Phases Cancer Center CLIP^2^, Marseille; 19 Gustave Roussy, Université Paris-Saclay, Villejuif; 20 Clinical Research Department, CLIP^2^ and ARPEGO Network, Rennes; 21 CLIP^2^ Lille, Centre Oscar Lambret, Lille and Lille University, Lille; 22 Lyon 1 University, EA4129, Lyon, France

**Keywords:** early-phase clinical trials, cancer, older patients, enrollment, access

## Abstract

**Background:**

Access to clinical trials and especially early-phase trials (ECT) is an important issue in geriatric oncology. As cancer can be considered an age-related disease because the incidence of most cancers increases with age, new drugs should also be evaluated in older patients to assess their safety and efficacy. The EGALICAN-2 study was primarily designed to identify social and/or regional inequalities regarding access to ECT. We focused on the factors of inequalities in access to ECT in older patients.

**Patients and methods:**

During a 1-year period (2015-2016), a survey was conducted in 11 early-phase units certified by the French National Cancer Institute.

**Results:**

A total of 1319 patients were included in the analyses: 1086 patients (82.3%) were <70 years and 233 patients (17.7%) were >70 years. The most common tumor types at referral in older patients were gastrointestinal (19.3%), hematological (19.3%), and thoracic tumors (18.0%). Most patients referred to the phase I unit had signed informed consent and the rate was similar across age (92.7% in younger patients versus 90.6% in older patients; *P* = 0.266). The rate of screening failure was also similar across age (28.5% in younger patients versus 24.3% in older patients; *P* = 0.219). Finally, in older patients, univariate analyses showed that initial care received in the hospital having a phase I unit was statistically associated with first study drug administration (odds ratio 0.49, 90% confidence interval 0.27-0.88; *P* = 0.045).

**Conclusions:**

Older patients are underrepresented in early clinical trials with 17.7% of patients aged ≥70 years compared with the number of new cases of cancer in France (50%). However, when invited to participate, older patients were prone to sign informed consent.

## Highlights

• Older patients are underrepresented in early-phase clinical trials (17.7%) compared with the number of new cases (50%).
• The rate of signed informed consent was similar across age groups (92.7% in younger patients versus 90.6% in older patients).
• The rate of screening failure was consistent across all age groups (28.5% in younger patients versus 24.3% in older patients).
• In older patients the initial care received in the center having a phase I unit was associated with study drug administration.

## Introduction

Cancer is the second most common cause of death in older adults after heart disease.^1^ The death rate from cancer in the United States in adults aged ≥85 years was expected to be ∼103 250 in 2019 (49 040 male and 54 210 female deaths), accounting for 17% of all cancer deaths.^1^ The number of new cancer cases is expected to rise rapidly over the next years due to the aging and growth of the population.^2^ However, older patients remain underrepresented in clinical trials despite published recommendations from the Food and Drug Administration (FDA) as early as 1989.^3^ In March 2020, new guidelines have been implemented with explicit recommendations for including an adequate representation of older adults in cancer clinical trials.^4^ They highlight the importance of enrolling patients, especially those aged ≥75, in early-phase clinical trials, and evaluate potential drug interactions early in the development process, to benefit older adults when designing trials and recruitment strategies; collecting data specific to older adults during trials and including older adults in postmarketing studies are also of major importance.^4^ In France, the identification and support of research centers specialized in early-phase clinical trials (ECT) were part of the 2009-2013 Cancer Plan. This initiative set up a powerful network over the French territory to ensure access to ECT regardless of the patient residence. The EGALICAN-2 study was primarily designed to identify social and/or regional inequalities regarding access to ECT. In this study, we focused on the access to ECT in the aging population.

## Methods

### EGALICAN-2 survey

EGALICAN-2 is a national multicenter prospective study designed to assess the equality of access to ECT. The study was approved by the French Advisory Committee on the Treatment of Information in Health Research (CCTIRS number: *13.660*) and by the French Data Protection Authority (CNIL number: *DR-2014-331*). During a 1-year period (2015-2016), a survey was conducted in 11 early-phase units certified by the French National Cancer Institute. All patients referred to the hospital were able to give consent to participate in the study and fulfill a survey developed by a sociologist team. The survey was divided in two parts: the first dedicated to the patient for personal information, the second, dedicated to the medical staff for patient medical information. We collected socioeconomical information (e.g. age, sex, place of birth, level of education, and profession), medical information (e.g. cancer site, type of treatment, and grade), and ECT information. The geographic context of the patients’ place of residence was considered using the French Deprivation Index^5^ as well as the patients’ travel time by car to the inclusion center. The higher the French Deprivation Index, the higher deprived the patient’s place of residence. The geographic context was not considered for patients from French overseas territories. The population-based cancer incidence estimates for the year 2020 were obtained for all cancers, in patients aged ≥70 years in France using the GLOBOCAN database produced by the International Agency for Research on Cancer (gco.iarc.fr).^6^

### Statistical analysis

Patients were divided into two categories according to age: an older group (≥70 years) and a younger group (<70 years). Qualitative and quantitative variables were described by their absolute and relative frequencies and mean (standard deviation) and median (minimum-maximum), respectively, and then compared using the χ^2^ or Fisher’s exact test and the Mann–Whitney nonparametric test, respectively. The percentage of older patients with cancer in the EGALICAN-2 study and the percentage of older patients with cancer in France were compared using a Z-test. Univariate logistic regression models were performed in older patients to explore factors associated with the first administration of the experimental treatment (cycle 1, day 1) and to estimate odds ratios (ORs) and 90% confidence intervals (CIs). All analyses were performed with SAS software (version 9.4; SAS Institute Inc., Cary, NC, USA) and Python (version 3.8.5).

## Results

### Patient characteristics at referral

**Figure 1:**
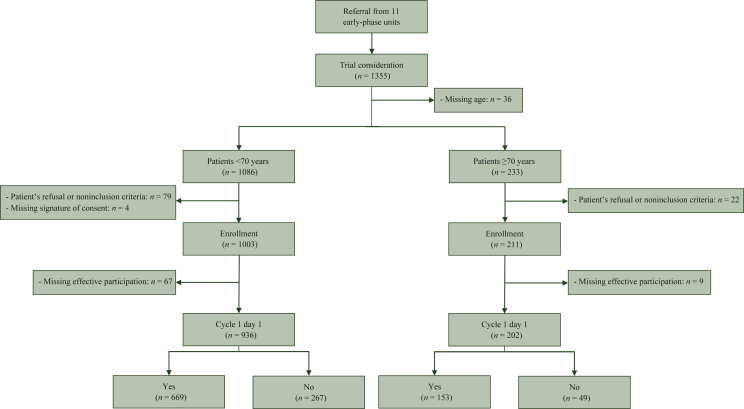
Flowchart of patients included in the analyses.

Between 2015 and 2016, a total of 1355 patients referred from 11 early-phase units in France participated in the study. Among them, 1319 patients (97.3%) had the information about age. Most patients referred in ECT were <70 years (*n* = 1086, 82.3%), and 233 patients (17.7%) were >70 years (Figure 1). The median age was 58 (range 17-70) years in the younger population and 74 (range 70-97) years in the older population. Significantly more female patients were referred among younger patients (55.5%) and male patients among older patients (52.4%; *P* = 0.028; Table 1). Most patients were treated for solid tumors but the proportion of patients treated for hematological malignancies was higher in older patients (19.3% versus 8.7%). Patients had predominantly metastatic cancer (*n* = 797, 87.9%; Table 2). There was no other difference when looking specifically in the population of patients aged ≥80 years (Supplementary Table S1, available at https://doi.org/10.1016/j.esmoop.2022.100468).

**Table 1:** Social and geographic characteristics of patients at referral according to their age

Characteristics	<70 years (*n* = 1086), *n* (%)	≥70 years (*n* = 233), *n* (%)	*P* value
*Social characteristics*			
**Age at the date of presentation of the trial (in years)**			
Mean (standard deviation)	55 (11.3)	75 (4.1)	
Median (range)	58 (17 to 70)	74 (70 to 97)	
**Sex**			0.028
Female	603 (55.5)	111 (47.6)	
Male	483 (44.5)	122 (52.4)	
**Place of residence outside France**			>0.99^a^
No	1081 (99.5)	232 (99.6)	
Yes	5 (0.5)	1 (0.4)	
**Place of birth**			0.718
Continental France	955 (88.3)	203 (87.5)	
Other	126 (11.7)	29 (12.5)	
**Number of people at home (including the patient)**			<0.001
1 person	159 (14.8)	53 (22.7)	
2 people	543 (50.4)	166 (71.2)	
≥3 people	375 (34.8)	14 (6.0)	
**Private insurance**			0.164
No	44 (4.1)	5 (2.2)	
Yes	1021 (95.9)	223 (97.8)	
**Education level**			<0.001
<High-school degree	498 (46.9)	137 (60.4)	
=High-school degree	175 (16.5)	36 (15.9)	
>High-school degree	388 (36.6)	54 (23.8)	
**Employment status**			<0.001
Active^b^	307 (30.4)	0 (0.0)	
Inactive^c^	704 69.6)	231 (100.0)	
**Health sector profession**			0.650
No	947 (91.0)	194 (91.9)	
Yes	94 (9.0)	17 (8.1)	
*Geographic characteristics*			
**French Deprivation Index**			0.167^d^
Mean (standard deviation)	−0.3 (1.6)	−0.4 (1.6)	
Median (range)	−0.2 (−6 to 6)	−0.4 (−6 to 5)	
**Journey time to the inclusion center (in minutes)**			0.006^b^
Mean (standard deviation)	104 (101.1)	86 (82.9)	
Median (range)	81 (0 to 1155)	66 (0 to 539)	

A chi-square test was used unless indicated otherwise.

a Fisher’s exact test.

b Full-time job and part-time job.

c Looking for employment, retired, at home, disability, training, medical leave, cessation of self-employed activity, no profession, and student.

d Mann–Whitney nonparametric test.

**Table 2:** Clinical characteristics of patients at referral according to their age

Characteristics	<70 years (*n* = 1086), *n* (%)	≥70 years (*n* = 233), *n* (%)	*P* value
**Cancer site**^a^			
Brain	44 (4.1)	4 (1.7)	
Breast	184 (17.0)	22 (9.4)	
Endocrine	17 (1.6)	2 (0.9)	
Gastrointestinal	235 (21.7)	45 (19.3)	
Gynecological	117 (10.8)	25 (10.7)	
Head and neck	79 (7.3)	9 (3.9)	
Respiratory system	148 (13.7)	42 (18.0)	
Skin	16 (1.5)	3 (1.3)	
Sarcoma	54 (5.0)	1 (0.4)	
Urological	88 (8.1)	35 (15.0)	
Blood	94 (8.7)	45 (19.3)	
Unknown	7 (0.6)	1 (0.4)	
**Drugs combination**			0.323
No	430 (48.6)	104 (52.5)	
Yes	454 (51.4)	94 (47.5)	
**Metastatic cancer**			0.744
No	93 (12.3)	17 (11.3)	
Yes	664 (87.7)	133 (88.7)	
**Initial care received in another hospital without a phase I unit**			0.183
No	366 (34.2)	89 (38.9)	
Yes	703 (65.8)	140 (61.1)	
**Signature of consent**			0.266
No	79 (7.3)	22 (9.4)	
Yes	1003 (92.7)	211 (90.6)	
**Reason for nonsignature of consent (*n* = 101)**			0.618^b^
Biological criteria	34 (53.1)	8 (38.1)	
No place available	10 (15.6)	5 (23.8)	
Patient	14 (21.9)	6 (28.6)	
Other	6 (9.4)	2 (9.5)	
**Administration of the experimental treatment (cycle 1 day 1, *n* = 1214)**			0.219
No	267 (28.5)	49 (24.3)	
Yes	669 (71.5)	153 (75.7)	

A chi-square test was used unless indicated otherwise.

a Total percentages may exceed 100 since several responses were possible.

b Fisher’s exact test.

Considering the estimated number of new cases in 2020, all cancers, both sexes, with an age of ≥20 years in France (465 125 cases), and the estimated number of new cases with an age of ≥70 years (232 294 cases), the number of new cases of cancer in older patients represented 50% of all cases in France. The difference between the rate of enrollment in our study (17.7%) compared with 50% (number of new cases considered as reference) was statistically significant with (*P* < 0.001).

### Signature of informed consent and first administration of the experimental treatment

Most patients that were referred to the phase I unit had signed informed consent. The rate was similar across age (92.7% in younger patients versus 90.6% in older patients; *P* = 0.266). In the older population, reasons for absence of signature were patient’s refusal to participate in the study (28.6%), exclusion biological criteria (38.1%), no slot available (23.8%), and others (9.5%).

The rate of screening failure was 28.5% in younger patients versus 24.3% in older patients (*P* = 0.219; Table 2). Reasons for screening failure are described for the whole population in Supplementary Table S2, available at https://doi.org/10.1016/j.esmoop.2022.100468. The main reasons were as follows: protocol-required tumor did not meet eligibility criteria (41.2%), development of an interval medical issue that precluded proceeding with study participation (12.2%), patient declined participation after signed consent (5.4%), discovery of an exclusionary preexisting medical condition (5.1%), out-of-protocol-specified range for chemical laboratory results (4.8%), imaging/radiology issue (3.7%), and presence of exclusionary brain metastasis (1.7%).

### Geographic/social access to innovative drugs

**Figure 2:**
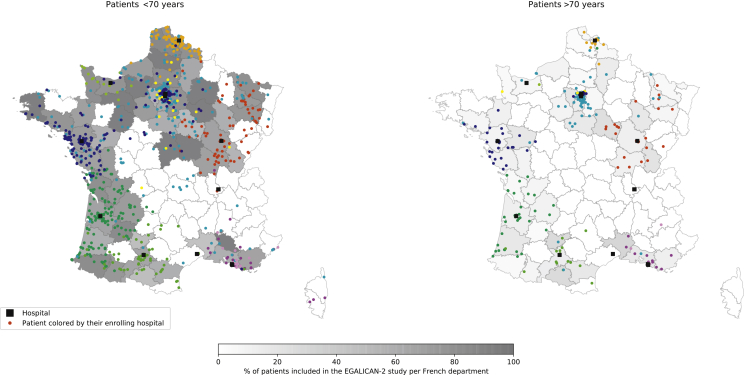
Patient referral in early-phase clinical trials per French department according to age.

Older patients who were referred to the phase I unit predominantly lived with one person (71.2%) compared with younger patients (50.4%; *P* < 0.001). They lived closer to the hospital compared with their younger counterparts [median 66 minutes by car (range 0-539) for patients older than 70 years versus 81 minutes by car (range 0-1155) for patients younger than 70 years; *P* = 0.006; Table 1]. The geographical distribution of referral of older patients in France is described on Figure 2.

Older patients had a significantly lower education level (<high-school degree) as compared with younger patients (60.4% versus 46.9%; *P* < 0.001; Table 1). There were more craftsmen/retailers/business leaders and executives/white-collar workers in the older population compared with younger patients (18.5% versus 10.9% and 28.7% versus 23.6% respectively; *P* = 0.016; Supplementary Figure S1, available at https://doi.org/10.1016/j.esmoop.2022.100468). There was no difference in terms of French Deprivation Index (*P* = 0.167). Other social characteristics are detailed in Table 1.

### Treatment characteristics

Targeted therapies and immunotherapy agents were evaluated in 40.1% and 30.6% of patients <70 years and 39.2% and 25.6% of patients >70 years, respectively (*P* = 0.001). Epigenetic drugs were used in 10.1% of older patients and in 6.5% of younger patients in relation to the higher number of older patients treated for hematological malignancies. Finally, hormonotherapy and chemotherapy were evaluated in 0.7% and 1.8% of younger patients and 4.8% and 3.1% of older patients (Supplementary Figure S2, available at https://doi.org/10.1016/j.esmoop.2022.100468). Older patients received combination therapy in 47.5% of cases compared with 51.4% in younger patients (*P* = 0.323; Table 2).

### Factors associated with first study drug administration in the older population

The univariate analyses showed that initial care received in the hospital having a phase I unit was statistically associated with the administration of the treatment (OR 0.49, 90% CI 0.27-0.88; *P* = 0.045). No other factor was significantly associated with first study drug administration (Table 3).

**Table 3:** Results of the univariate logistic regression of factors associated with the administration of the experimental treatment in patients older than 70 years

Factors	Administration of the experimental treatment (cycle 1 day 1, *n* = 202)		OR (90% CI)	*P* value
	Yes, *n* (%)	No, *n* (%)		
**Sex**				0.822
Female	69 (75.00)	23 (25.00)	1	
Male	84 (76.36)	26 (23.64)	1.08 (0.63-1.85)	
**Drugs combination**				0.404
No	63 (72.41)	24 (27.59)	1	
Yes	67 (77.91)	19 (22.09)	1.34 (0.75-2.40)	
**Number of people at home (including the patient)**				0.253
1 people	37 (82.22)	8 (17.78)	1	
≥3 people	116 (73.89)	41 (26.11)	0.61 (0.30-1.24)	
**Education level**				0.670
<High-school degree	89 (76.72)	27 (23.28)	1	
≥High-school degree	60 (74.07)	21 (25.93)	0.87 (0.50-1.51)	
**Initial care received in another hospital without a phase I unit**				0.045
No	69 (83.13)	14 (16.87)	1	
Yes	82 (70.69)	34 (29.31)	0.49 (0.27-0.88)	

OR, odds ratio; 90% CI, 90% confidence interval.

## Discussion

To our knowledge, this is the first study to report a national experience of access to innovative drugs for older patients. The expansion of early clinical trials and geriatric oncology have been a priority across all French Cancer plans since 2006 and 2009. However, older patients remain underrepresented in clinical trials, and especially in early-phase clinical trials, even though they represent 42% of the overall cancer population.^7^, ^8^, ^9^, ^10^, ^11^ In our analysis, they represented 17.7% of the total number of patients enrolled, whereas in France they represent 50% of new cases of cancer (gco.iarc.fr). A recent study found that the median age of patients, in more than 300 randomized trials involving four common cancers, was 6.5 years younger than that of the general population with the same diagnoses.^11^ Many potential barriers have been identified related to patient, physician, trial/protocol, and logistics.^12^ One of the first potential barrier is age alone. In the last years there has been a tremendous effort to avoid age as an exclusion criterion. In a recent study, Ludmir et al.^11^ found that upper age restriction criterion was identified in 10.1% of 742 phase III randomized clinical trials. Phase I trials were not included in the analysis. Alternate eligibility criteria that can exclude older patients are related to functional status, organ function, comorbidity, and comedication that are frequent in the older population. These criteria may vary according to the type of compound tested (e.g. less restrictive with immune checkpoint blockers or immunotherapy compared with chemotherapy clinical trials). However, these are often included systematically in protocols with few scientific background.^13^^,^^14^ American Society of Clinical Oncology (ASCO), Friends of Cancer Research, and the FDA published suggestions to revise some of these criteria to avoid too many restrictive criteria and improve patient accrual in clinical trials.^4^^,^^15^^,^^16^ The European Medicines Agency (EMA) also issued guidance on geriatric medicine strategies in 2011 to ensure that the needs of older people are taken into account in the development and evaluation of new medicines.^17^^,^^18^ Most of the data available in older patients are derived from a subgroup analyses of phase III registration trials, making it difficult to extrapolate. Moreover, the older population is very heterogeneous and chronological age itself does not reflect physiological age assessed by comprehensive geriatric assessment.^19^ Comprehensive geriatric assessment explores different areas including functional status, mobility, cognition, emotional status, nutritional status, comorbidities, polypharmacy, and social support. It helps guiding treatment decision and implementing interventions to improve patients outcome.^20^, ^21^, ^22^, ^23^ Its use is recommended by international societies.^24^^,^^25^ Geriatric assessment is not yet implemented in clinical trials despite the critical need to provide data to better describe the older population enrolled in clinical trials. To allow both a minimal geriatric description of the older patients with cancer and a standardization of geriatric data, some tools have been developed such as the Geriatric Core Dataset (G-CODE) specifically designed for clinical trials.^26^ It consists of two social questions, two autonomy scales (activities of daily living and 4-item instrumental activities of daily living), a mobility scale (Timed Get Up and Go Test), two nutrition items (weight loss and body mass index), a cognitive scale (Mini-Cog), a scale assessing the depressive mood (Mini-Geriatric Depression Scale), and a comorbidity overview (updated Charlson Comorbidity Index).^26^

Reports from phase I units in cancer centers worldwide have not shown increased toxicity or dose-limiting toxicities with the exception of Schwandt et al., who described an association between age >80 years and the probability of dose-limiting toxicity.^27^, ^28^, ^29^, ^30^, ^31^, ^32^, ^33^, ^34^, ^35^, ^36^, ^37^ Therefore enrollment of older patients in early-phase clinical trials should be encouraged by health authorities not only in the United States but also in Europe. Le Saux et al.^38^ showed that efforts have been made comparing two periods (2001-2004 and 2011-2014). There was an increase in the number of clinical trials reporting results on older patients, especially dedicated phase I studies and subgroup analyses of phase III studies.^38^ However, no trials required an assessment of physiological age at study entry to help physician decision in real life.

In our study, older patients lived closer to the hospital. This is consistent with previous reports. Basche et al.^39^ found that traveling to the University Cancer Center was the most frequent barrier to participate in early-phase trials (34% of respondents; 95% CI 29%-38%). In another study conducted as semistructured interviews of community and academic oncologists, one of the most common barriers cited by community oncologists was caregiver burden (12% of participants interviewed), including emotional and logistical barriers that can influence patient willingness to participate.^40^ One of the proposals made in an FDA-ASCO workshop was to work with sponsors to open more trials in community settings.^15^ However, phase I units usually require specific facilities that may not be available in community centers.

Surprisingly, the sex ratio was in favor of males in older patients and females in younger patients. This might be explained by the different tumor types in each cohort. There were more patients with breast cancer in the younger population and more patients with hematological malignancies in the older population.

Signature of informed consent was similar between the two age categories as well as the rate of screening failure. The proportion of screening failure was consistent with that found in the published literature. In a monocentric retrospective study performed among 773 American patients with cancer included in phase I trials, one-quarter of them were screening failures.^41^ Another French case–control monocentric study showed that among 1293 patients enrolled in phase I trials, 15% were screening failures.^42^ Our results support the evidence that older patients are not at an increased risk of screening failure and therefore should not be excluded from early-phase clinical trials.

Education level was not associated in univariate analysis with the administration of the experimental treatment in older patients. This is an encouraging result suggesting that level of education did not affect participation in an early clinical trial. This is not consistent with a previous report that showed that patients with lower incomes were less likely to participate in a clinical trial.^43^^,^^44^ However, this difference might be related to the health care system. Patients with a higher-income in the United States are better insured. In France, patients are entitled to public health insurance regardless of their income. In our study, all patients were seen in the hospital by the phase I team to discuss the clinical trial. They were already selected by their caring physician to potentially fulfill clinical trial inclusion criteria. Mohd Noor et al.^44^ showed in a multivariate analysis that most deprived patients were significantly less likely to be referred for consideration of an early-phase clinical trial [OR 0.53, 95% CI 0.38-0.74; *P* = 0.002). This difference was lost once referred for consideration (OR 0.81, 95% CI 0.40-1.63; *P* = 0.163). This might explain our results.

Our findings must be interpreted in the light of some limitations. First, the survey was conducted between 2015 and 2016 before the immunotherapy era. One of the main barriers to enrollment stated by older patients is the concerns about moderate or severe toxicity.^39^ Immunotherapy is an attractive option in older patients with fewer toxicities than cytotoxic chemotherapies or some targeted therapies and might influence the enrollment in clinical trials.^45^ Second, the survey did not include all phase I units in France. However, 11 units agreed to participate out of a total of 16 units in France (68.8%). It would be of particular interest to reiterate the survey a few years later to see potential changes in the enrollment of older patients.

Considering the epidemiological changes and the growing population of older patients with cancer in the future, enrollment of this population remains a major concern.^46^ Health authorities should follow the footsteps of the FDA and engage more efforts in the assessment of treatment efficacy and safety in older patients. Along with these measures, some specificities related to older patients should be considered and implemented to increase enrollment in early-phase clinical trials such as dedicated patient care, tailored information, and early involvement of the caregiver.

### Conclusions

Older patients are underrepresented in early clinical trials with 17.7% of patients aged ≥70 years in our study compared with the number of new cases of cancer in France (50%). However, when invited to participate, older patients were prone to sign informed consent.
