# Supplementary material for: Access to early-phase clinical trials in older patients with cancer in France: the EGALICAN-2 study
Source: ESMO Open. 2022 May 6;7(3):100468. doi: 10.1016/j.esmoop.2022.100468 (PMC9271476; doi:10.1016/j.esmoop.2022.100468)
Supplement: Supplementary Figures S1 and S2 [file mmc1.pdf]

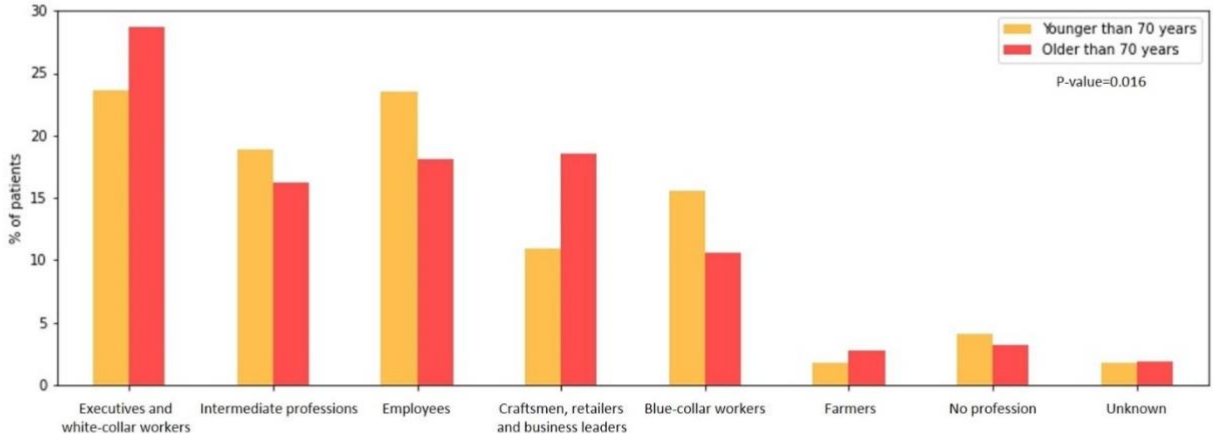

Figure 1. Professions and socio-professional categories according to age. A Fisher's exact test is used.

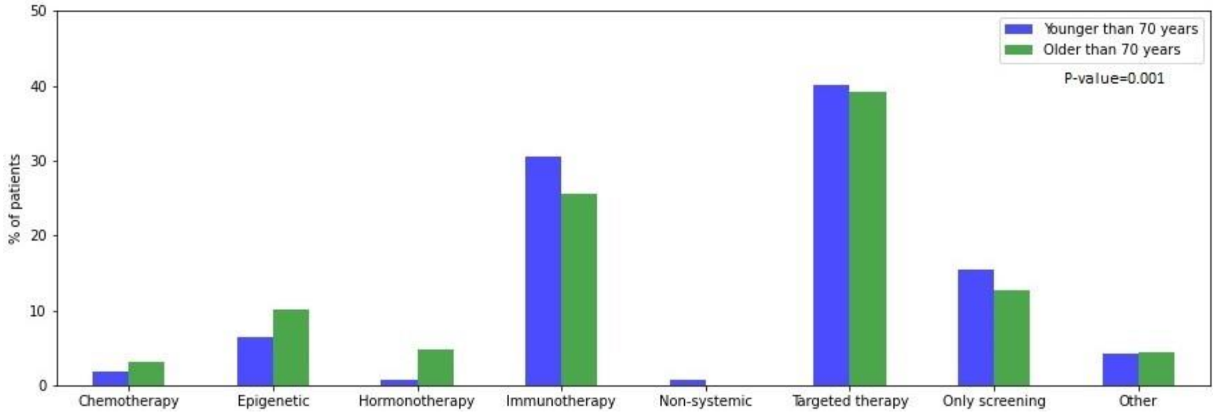

Figure 2. Type of treatment according to age. A Fisher's exact test is used.
