# Supplementary material for: Access to early-phase clinical trials in older patients with cancer in France: the EGALICAN-2 study
Source: ESMO Open. 2022 May 6;7(3):100468. doi: 10.1016/j.esmoop.2022.100468 (PMC9271476; doi:10.1016/j.esmoop.2022.100468)
Supplement: Supplementary Tables S1 and S2 [file mmc2.docx]

***Table 1 Supplementary.*** *Patients characteristics according to their age (3 groups).*

|  | **[17-70 years[**  **(n=1 086)** | | **[70-80 years[ (n=207)** | | **≥ 80 years (n=26)** | |  |
| --- | --- | --- | --- | --- | --- | --- | --- |
|  | **n** | **%** | **n** | **%** | **n** | **%** | **P-value** |
| **Age at the date of presentation of the trial (in years)** |  | |  | |  | |  |
| Mean (standard deviation) | 55 (11.3) | | 74 (2.6) | | 84 (3.5) | |  |
| Median (range) | 58 (17;70) | | 74 (70;80) | | 83 (80;97) | |  |
|  |  |  |  |  |  |  |  |
| **Gender** |  |  |  |  |  |  | 0.089 |
| Female | 603 | 55.5 | 99 | 47.8 | 12 | 46.2 |  |
| Male | 483 | 44.5 | 108 | 52.2 | 14 | 53.8 |  |
| **Place of residence outside France** |  |  |  |  |  |  | 1.000* |
| No | 1081 | 99.5 | 206 | 99.5 | 26 | 100.0 |  |
| Yes | 5 | 0.5 | 1 | 0.5 | 0 | 0.0 |  |
| **Cancer site^1^** |  |  |  |  |  |  |  |
| Skin | 16 | 1.5 | 2 | 1.0 | 1 | 3.8 |  |
| Head and neck | 79 | 7.3 | 8 | 3.9 | 1 | 3.8 |  |
| Sarcoma | 54 | 5.0 | 1 | 0.5 | 0 | 0.0 |  |
| Gastrointestinal | 235 | 21.7 | 43 | 20.8 | 2 | 7.7 |  |
| Brain | 44 | 4.1 | 4 | 1.9 | 0 | 0.0 |  |
| Blood | 94 | 8.7 | 41 | 19.8 | 4 | 15.4 |  |
| Breast | 184 | 17.0 | 17 | 8.2 | 5 | 19.2 |  |
| Endocrine | 17 | 1.6 | 2 | 1.0 | 0 | 0.0 |  |
| Gynecological | 117 | 10.8 | 24 | 11.6 | 1 | 3.8 |  |
| Respiratory system | 148 | 13.7 | 39 | 18.8 | 3 | 11.5 |  |
| Urological | 88 | 8.1 | 26 | 12.6 | 9 | 34.6 |  |
| Unknown | 7 | 0.6 | 1 | 0.5 | 0 | 0.0 |  |
| Missing | 3 |  | 0 |  | 0 |  |  |
| **Drugs combination** |  |  |  |  |  |  | 0.158 |
| No | 430 | 48.6 | 90 | 50.6 | 14 | 70.0 |  |
| Yes | 454 | 51.4 | 88 | 49.4 | 6 | 30.0 |  |
| Not applicable (only screening) | 161 |  | 23 |  | 6 |  |  |
| Missing | 41 |  | 6 |  | 0 |  |  |
| **Metastatic cancer** |  |  |  |  |  |  | 0.968* |
| No | 93 | 12.3 | 15 | 11.5 | 2 | 10.5 |  |
| Yes | 664 | 87.7 | 116 | 88.5 | 17 | 89.5 |  |
| Missing | 329 |  | 76 |  | 7 |  |  |
| **Grade at the date of presentation of the trial to the patient** |  |  |  |  |  |  | 0.597* |
| Grade 1 | 7 | 6.7 | 2 | 9.1 | 0 | 0.0 |  |
| Grade 2 | 44 | 41.9 | 9 | 40.9 | 0 | 0.0 |  |
| Grade 3 | 38 | 36.2 | 7 | 31.8 | 0 | 0.0 |  |
| Grade 4 | 16 | 15.2 | 4 | 18.2 | 1 | 100.0 |  |
| Missing | 981 |  | 185 |  | 25 |  |  |
| **Signature of consent** |  |  |  |  |  |  | 0.083* |
| No | 79 | 7.3 | 17 | 8.2 | 5 | 19.2 |  |
| Yes | 1003 | 92.7 | 190 | 91.8 | 21 | 80.8 |  |
| Missing | 4 |  | 0 |  | 0 |  |  |
| **Reason for non-signature of consent (n=101)** |  |  |  |  |  |  | 0.383* |
| Biological criteria | 34 | 53.1 | 6 | 37.5 | 2 | 40.0 |  |
| No place available | 10 | 15.6 | 5 | 31.3 | 0 | 0.0 |  |
| Patient | 14 | 21.9 | 3 | 18.8 | 3 | 60.0 |  |
| Other | 6 | 9.4 | 2 | 12.5 | 0 | 0.0 |  |
| Missing | 15 |  | 1 |  | 0 |  |  |
| **Administration of the experimental treatment (cycle 1 day 1, n=1 214)** |  |  |  |  |  |  | 0.425 |
| No | 267 | 28.5 | 45 | 24.7 | 4 | 20.0 |  |
| Yes | 669 | 71.5 | 137 | 75.3 | 16 | 80.0 |  |
| Missing | 67 |  | 8 |  | 1 |  |  |
| **Immediate presentation of the trial** |  |  |  |  |  |  | **0.027** |
| No | 712 | 65.8 | 147 | 71.0 | 22 | 88.0 |  |
| Yes | 370 | 34.2 | 60 | 29.0 | 3 | 12.0 |  |
| Missing | 4 |  | 0 |  | 1 |  |  |
| **Place of birth** |  |  |  |  |  |  | 0.344* |
| Continental France | 955 | 88.3 | 178 | 86.4 | 25 | 96.2 |  |
| Other | 126 | 11.7 | 28 | 13.6 | 1 | 3.8 |  |
| Missing | 5 |  | 1 |  | 0 |  |  |
| **Number of people at home (including the patient)** |  |  |  |  |  |  | **< 0.001*** |
| 1 person | 159 | 14.8 | 45 | 21.7 | 8 | 30.8 |  |
| 2 people | 543 | 50.4 | 149 | 72.0 | 17 | 65.4 |  |
| ≥ 3 people | 375 | 34.8 | 13 | 6.3 | 1 | 3.8 |  |
| Missing | 9 |  | 0 |  | 0 |  |  |
| **Private insurance** |  |  |  |  |  |  | 0.451* |
| No | 44 | 4.1 | 5 | 2.5 | 0 | 0.0 |  |
| Yes | 1021 | 95.9 | 198 | 97.5 | 25 | 100.0 |  |
| Missing | 21 |  | 4 |  | 1 |  |  |
| **Education level** |  |  |  |  |  |  | **0.001*** |
| < High school degree | 498 | 46.9 | 121 | 60.2 | 16 | 61.5 |  |
| = High school degree | 175 | 16.5 | 30 | 14.9 | 6 | 23.1 |  |
| > High school degree | 388 | 36.6 | 50 | 24.9 | 4 | 15.4 |  |
| Missing | 25 |  | 6 |  | 0 |  |  |
| **Employment status** |  |  |  |  |  |  | **< 0.001** |
| Active^2^ | 307 | 30.4 | 0 | 0.0 | 0 | 0.0 |  |
| Inactive^3^ | 704 | 69.6 | 205 | 100.0 | 26 | 100.0 |  |
| Missing | 75 |  | 2 |  | 0 |  |  |
| **Health sector profession** |  |  |  |  |  |  | 0.198* |
| No | 947 | 91.0 | 175 | 93.1 | 19 | 82.6 |  |
| Yes | 94 | 9.0 | 13 | 6.9 | 4 | 17.4 |  |
| Missing | 45 |  | 19 |  | 3 |  |  |
| **Initial care received in another hospital without a phase I unit** |  |  |  |  |  |  | 0.142 |
| No | 366 | 34.2 | 76 | 37.3 | 13 | 52.0 |  |
| Yes | 703 | 65.8 | 128 | 62.7 | 12 | 48.0 |  |
| Missing | 17 |  | 3 |  | 1 |  |  |
| **Patient care pathway** |  |  |  |  |  |  | 0.136 |
| Internal to the inclusion centre | 366 | 34.3 | 76 | 37.3 | 13 | 52.0 |  |
| External-internal to the inclusion centre | 368 | 34.5 | 75 | 36.8 | 9 | 36.0 |  |
| External to the inclusion centre | 332 | 31.1 | 53 | 26.0 | 3 | 12.0 |  |
| Missing | 20 |  | 3 |  | 1 |  |  |
| **French deprivation index** |  |  |  |  |  |  | 0.352** |
| Mean (standard deviation) | -0.3 (1.6) | | -0.5 (1.6) | | -0.2 (1.7) | |  |
| Median (range) | -0.2 (-6;6) | | -0.4 (-6;5) | | -0.4 (-4;4) | |  |
| Missing | 11 | | 2 | | 0 | |  |
| **Journey time to the inclusion centre (in minutes)** |  |  |  |  |  |  | **0.021**** |
| Mean (standard deviation) | 104 (101.1) | | 86 (81.6) | | 86 (93.6) | |  |
| Median (range) | 81 (0;1 155) | | 66 (0;539) | | 74 (0;477) | |  |
| Missing | 11 | | 2 | | 0 | |  |

A χ^2^ is used unless indicated otherwise; *Fisher’s exact test; **Kruskal-Wallis test; ^1^Total percentages may exceed 100 since several responses were possible; ^2^Full-time job and part-time job; ^3^Looking for employment, retired, at home, disability, training, medical leave, cessation of self-employed activity, no profession and student.

***Table 2 Supplementary.*** *Reasons for screening failure for the whole population (n=316).*

| **Screening failure reasons** | **n** | **%** |
| --- | --- | --- |
| Abnormal EKG findings | 1 | 0.3 |
| Development of an interval medical issue that precluded proceeding with study participation | 36 | 12.2 |
| Discovery of an exclusionary pre-existing medical condition | 15 | 5.1 |
| Imaging/radiology issue | 11 | 3.7 |
| Multiple reasons | 3 | 1.0 |
| Out-of-protocol-specified range for chemistry laboratory results | 14 | 4.8 |
| Out-of-protocol-specified range for hematology laboratory results | 3 | 1.0 |
| Presence of exclusionary brain metastasis | 5 | 1.7 |
| Protocol required tumor or host analyte did not meet eligibility criteria | 121 | 41.2 |
| Slot non-available | 3 | 1.0 |
| Study-specific special exam issue | 1 | 0.3 |
| Subject declined participation after signed consent | 16 | 5.4 |
| Other | 65 | 22.1 |
